# Baicalein Synergizes with Docetaxel in TNBC and the Combination Suppresses AKT/GSK3β/β-Catenin Signaling

**DOI:** 10.3390/cimb48101066

**Published:** 2026-10-15

**Authors:** Taomei Yang, Xiangyun Chen, Chunwei Chu, Yonghu Wan

**Affiliations:** 1 School of Basic Medicine, Guizhou University of Traditional Chinese Medicine, Guiyang 550025, China; yangtaomei017@gzy.edu.cn (T.Y.);; 2 Guizhou Center for Disease Control and Prevention, Guiyang 550004, China

**Keywords:** baicalein, docetaxel, triple-negative breast cancer, AKT/GSK3β/β-catenin, HSP90AA1, drug synergy

## Abstract

Limited docetaxel efficacy in triple-negative breast cancer (TNBC) necessitates chemosensitizers. Baicalein is a potential chemosensitizer, but its synergy with docetaxel in TNBC is unclear. Here, we investigated the combined effects and underlying mechanisms. The concentrations of baicalein (80 μM) and docetaxel (20 nM) were tested on TNBC cells; synergy was assessed by CCK-8 and Chou–Talalay analysis. Proliferation, migration, invasion, and apoptosis were assessed by colony formation, wound healing, Transwell, Matrigel, and flow cytometry assays. Protein levels of p-AKT, p-GSK3β and β-catenin were determined by western blot, and HSP90AA1 mRNA by qPCR. Baicalein–HSP90AA1 binding was analyzed by molecular docking. The prognostic value of HSP90AA1 was analyzed by Kaplan–Meier Plotter. The combination exhibited synergistic anti-tumor activity (CI < 0.9), significantly suppressed proliferation, migration and invasion, and enhanced apoptosis. Mechanistically, the combination reduced p-AKT, p-GSK3β, and β-catenin protein levels without affecting HSP90AA1 mRNA. Molecular docking indicated a potential interaction between baicalein and HSP90AA1. High HSP90AA1 expression correlated with shorter distant metastasis-free survival (HR = 1.71, *p* = 0.0046) and worse overall survival (HR = 2.15, *p* = 0.021). Thus, baicalein synergizes with docetaxel to suppress TNBC in vitro, accompanied by suppression of AKT/GSK3β/β-catenin signaling, potentially through interaction with HSP90AA1, a proposed upstream target.

## 1. Introduction

Breast cancer remains the most common malignancy among women worldwide, with an estimated 2.3 million new cases in 2022 [1,2]. Triple-negative breast cancer (TNBC), characterized by the absence of estrogen receptor (ER), progesterone receptor (PR), and human epidermal growth factor receptor 2 (HER2), accounts for approximately 15–20% of all breast cancers but is responsible for a disproportionately high number of deaths. This is largely due to its aggressive behavior, high metastatic potential, and lack of effective targeted therapies [3,4].

Docetaxel is a semisynthetic taxane and commonly used as a chemotherapeutic agent for TNBC. It exerts its antitumor effects by binding to β-tubulin, promoting microtubule polymerization, and blocking cell cycle progression at the G2/M phase, ultimately inducing apoptosis [5,6,7]. Despite its clinical utility, docetaxel-based chemotherapy faces two major challenges: (i) intrinsic or acquired chemoresistance [6], often mediated by drug efflux pumps, β-tubulin isoform switching, and activation of survival pathways such as the AKT signaling pathway [8,9]; and (ii) severe dose-limiting toxicities, including neutropenia and peripheral neuropathy, which reduce patient compliance and treatment efficacy [6,9]. Therefore, identifying safe and effective sensitizers that can enhance docetaxel efficacy while reducing its adverse effects is of great clinical importance.

Baicalein is a bioactive flavonoid isolated from the root of *Scutellaria baicalensis* Georgi [10]. Pharmacological studies have demonstrated that baicalein possesses broad anticancer activities, including induction of apoptosis, inhibition of proliferation, migration, and invasion in various cancer types [10,11,12]. Notably, recent evidence suggests that baicalein can sensitize cancer cells to chemotherapeutic agents. In MCF-7 breast cancer cells, baicalein enhanced docetaxel-induced cytotoxicity and pro-apoptotic activity [13]. However, whether this sensitizing effect exists in the more aggressive TNBC subtype remains unexplored, and the underlying molecular mechanism is yet to be elucidated.

The AKT/GSK3β/β-catenin signaling axis is a well-established oncogenic pathway in TNBC [14,15,16]. Activated AKT phosphorylates GSK3β at Ser9, increasing p-GSK3β levels and inactivating GSK3β. This in turn reduces β-catenin degradation, leading to its stabilization and nuclear translocation [17], which drives the transcription of genes involved in proliferation, survival, and metastasis [14,18,19,20]. Aberrant activation of this axis is associated with chemoresistance and poor prognosis in TNBC [21,22]. Baicalein has been reported to modulate components of this pathway, including AKT and β-catenin [23,24], but whether it synergizes with docetaxel to suppress the entire axis in TNBC cells remains unknown.

An upstream regulator of this pathway is HSP90AA1 (heat shock protein 90 alpha family class A member 1). HSP90AA1 is a core ATP-dependent molecular chaperone whose function is driven by its ATP-dependent conformational cycle rather than by changes in its own expression level [25,26]. ATP binding induces a closed conformation that enables HSP90AA1 to stabilize key oncogenic client proteins, including AKT, EGFR, HER2, c-RAF, CDK4, HIF-1α, β-catenin, and hormone receptors [27,28], whereas ATP-competitive inhibitors disrupt this cycle, thereby compromising client protein stability and activity. Through this chaperone function, HSP90AA1 acts as a master regulator of malignant phenotypes, therapeutic resistance, and tumor plasticity [27,29]. Notably, inhibition of HSP90AA1 function compromises the stability and activity of its clients, thereby systematically disrupting tumor survival signaling [29]. Although HSP90 inhibitors have shown preclinical promise, over 20 compounds entering clinical trials over the past two decades have failed to gain FDA approval, primarily due to dose-limiting toxicities and paradoxical activation of the HSF1-driven heat shock response. This has prompted interest in natural-product-derived HSP90AA1 ligands, which may offer distinct pharmacological profiles. Importantly, baicalein has been shown to directly bind to HSP90AA1, leading to disrupted HSP90AA1–AKT binding and reduced AKT phosphorylation [30]. Given that AKT is a crucial client protein of HSP90AA1 and serves as a central node in the AKT/GSK3β/β-catenin signaling pathway, we hypothesized that baicalein synergizes with docetaxel to suppress TNBC malignancy by targeting HSP90AA1, thereby inhibiting AKT activity and subsequently inactivating the AKT/GSK3β/β-catenin pathway, leading to inhibition of proliferation, migration, and induction of apoptosis.

To test this hypothesis, we first evaluated the combined effects of baicalein and docetaxel on the malignant phenotypes of TNBC cells, including proliferation, migration, invasion, and apoptosis. We then explored the underlying mechanism by examining the AKT/GSK3β/β-catenin signaling axis and the potential involvement of HSP90AA1. Molecular docking was performed to explore the potential binding between baicalein and HSP90AA1. The prognostic value of HSP90AA1 was evaluated using publicly available TNBC patient datasets. Collectively, our findings provide functional and mechanistic evidence and prognostic insights regarding the baicalein–docetaxel combination in TNBC.

## 2. Materials and Methods

### 2.1. Reagents and Antibodies

Baicalein (purity ≧ 99%, cat. no. s2268) and docetaxel (purity ≥ 99%, cat. no. s1148) were obtained from Selleckchem (Selleck Chemicals, Houston, TX, USA). Baicalein and docetaxel were dissolved in DMSO. After dilution into complete culture medium, no precipitate was observed at the highest tested concentration (400 µM baicalein) under a light microscope. The CCK-8 kit was purchased from Beyotime (Cell Counting Kit-8, cat. no. C0038, Shanghai, China). The Annexin V-APC/7-AAD apoptosis detection kit was purchased from KeyGEN BioTECH (cat. no. KGA1026, Nanjing, China). Matrigel matrix (cat. no. 356234) and Transwell chambers (8.0 μm pore size, cat. no. 3422) were purchased from Corning (Corning, NY, USA). Anti-p-AKT (Ser473) was purchased from Cell Signaling Technology (CST, 4060T, Danvers, MA, USA), anti-p-GSK3β (Ser9) was purchased from Affinity (Affinity, AF2016, Changzhou, China), and GAPDH was purchased from Proteintech (GAPDH Monoclonal antibody, 60004-1-Ig, Wuhan, China). Anti-β-catenin was purchased from Affinity (Affinity, AF6266, Changzhou, China). TRIzol reagent and SYBR Green qPCR kits were purchased from Thermo Fisher Scientific (Waltham, MA, USA). All cell culture plates were purchased from Corning (Corning, NY, USA). The BCA Protein Assay Kit was purchased from GBCBIO (cat. no. G3522, Guangzhou, China). RIPA Lysis Buffer was purchased from Meilunbio (cat. no. MA0151, Dalian, China). Polyvinylidene difluoride (PVDF) membranes were purchased from Millipore (cat. no. IPVH00010, Burlington, MA, USA). Quantitative real-time PCR (qPCR) related reagents were purchased from VAZYME (Nanjing, China).

### 2.2. Cell Culture

The MDA-MB-231 and BT-549 human triple-negative breast cancer cells were purchased from Procell (CL-0150, CL-0041, Wuhan, China) and authenticated by STR profiling, which confirmed their identity and absence of cross-contamination. MDA-MB-231 cells were cultured in DMEM (Procell, Wuhan, China) supplemented with 10% fetal bovine serum (Excell Bio, Shanghai, China), 100 U/mL penicillin, and 100 μg/mL streptomycin (Aladdin, Shanghai, China). BT-549 cells were cultured in RPMI-1640 (Procell, Wuhan, China) supplemented with 10% fetal bovine serum, 100 U/mL penicillin, and 100 μg/mL streptomycin. Both cell lines were maintained at 37 °C in a humidified atmosphere containing 5% CO_2_. For experiments, cells were assigned to four groups: DMSO (vehicle control), baicalein (80 μM), docetaxel (20 nM), and their combination (baicalein 80 μM + docetaxel 20 nM).

### 2.3. CCK-8 Assay and IC50 Determination

Cells were seeded in 96-well plates at 8 × 10^3^ cells/well. Peripheral wells received 100 μL of sterile PBS to minimize edge effects. Blank wells containing only medium served as background controls. After overnight incubation at 37 °C to allow cell attachment, the cells were treated with various concentrations of baicalein (0, 1, 5, 10, 20, 40, 60, 80, 100, and 120 μM) or docetaxel (0, 0.2, 0.63, 2, 6.3, 20, 63, 200, and 400 nM) for 24 h. Then, 10 μL of CCK-8 reagent was added to each well and incubated for 2 h. Absorbance was measured at 450 nm using a microplate reader. The inhibition rate was calculated, and IC50 values were determined using GraphPad Prism 11. For combination experiments, sub-inhibitory concentrations were selected based on preliminary dose–response assays: baicalein 80 μM (24.53% inhibition) and docetaxel 20 nM (37.71% inhibition). The synergistic effect was preliminarily evaluated using Jin’s Q-value method, with Q > 1.15 defined as synergy [31]. Experiments were performed in triplicate wells and repeated at least three times independently.

### 2.4. Pharmacological Assessment of Drug Interactions Using the Chou–Talalay Method

The pharmacological interaction between baicalein and docetaxel was further evaluated using the Chou–Talalay method. In the Chou–Talalay analysis, a cell-free control containing 400 µM baicalein and CCK-8 reagent was compared with blank wells containing only medium and CCK-8. No significant difference was observed between the two groups. Consistently with Section 2.3, background correction was performed using blank wells. Drugs were prepared as described in Section 2.1. The final DMSO concentration was 0.2% in all treatment groups. Based on our previous Jin’s Q-value analysis, the combination of 80 μM baicalein and 20 nM docetaxel was selected as the reference combination. Drug combinations were then prepared at a fixed numerical ratio of 4:1, defined as baicalein concentration in μM to docetaxel concentration in nM. This numerical ratio corresponds to a molar ratio of 4000:1 (e.g., 80 μM baicalein = 80,000 nM; 80,000 nM: 20 nM = 4000:1). The fixed-ratio combination series comprised 4 μM baicalein + 1 nM docetaxel, 10 μM baicalein + 2.5 nM docetaxel, 20 μM baicalein + 5 nM docetaxel, 40 μM baicalein + 10 nM docetaxel, 80 μM baicalein + 20 nM docetaxel, 160 μM baicalein + 40 nM docetaxel, 320 μM baicalein + 80 nM docetaxel, and 400 μM baicalein + 100 nM docetaxel. These paired concentrations corresponded to 0.05-, 0.125-, 0.25-, 0.5-, 1-, 2-, 4-, and 5-fold multiples of the reference combination. The same fixed-ratio series was evaluated in both MDA-MB-231 and BT-549 cells to permit comparison of cell line-dependent interaction profiles.

Cell-viability data obtained from the CCK-8 assay were converted to fraction-affected values (Fa = 1 − relative cell viability), in which relative cell viability was background-corrected (using blank wells) and normalized to the DMSO vehicle control. Fa values from three independent experiments were averaged at each concentration and entered into CompuSyn (version 1.0) (ComboSyn, Inc., Paramus, NJ, USA) to generate fitted dose–effect curves and median-effect plots. The median-effect dose (Dm), slope of the median-effect plot (m), and linear correlation coefficient (r) were derived for each drug and cell model (MDA-MB-231 and BT-549). Combination index (CI) values were calculated for each independent experiment from the fitted median-effect model across multiple effect levels (Fa) and are reported as mean ± SD. These effect levels were derived from the fitted median-effect model and did not represent independently administered concentrations. Dose reduction index (DRI) for docetaxel was evaluated at Fa = 0.50 for each independent experiment and reported as mean ± SD, as model-derived estimates of the dose ratio between the single-agent and combination doses predicted to produce the same effect.

The pharmacological interaction was interpreted according to the predefined Chou–Talalay criteria: CI values <0.90 indicated synergism, values from 0.90 to 1.10 indicated an additive interaction, and values >1.10 indicated antagonism. DRI values were interpreted descriptively and were not considered evidence of clinical dose reduction or therapeutic benefit.

### 2.5. Colony Formation Assay

Cells were seeded into 6-well plates at a density of 500 cells/well and allowed to attach overnight. They were then treated with control (DMSO), baicalein (80 μM), docetaxel (20 nM), or their combination. The medium was replaced every 3 days. After two weeks, colonies were fixed with 75% ice-cold ethanol for 30 min. After removal of the fixative, 0.5% crystal violet was added to stain the colonies for 10–30 min. The colonies were rinsed with distilled water and air-dried. Each well was photographed separately using a digital camera, and colonies containing ≥50 cells were counted. The colony formation rate was calculated as follows: colony formation rate (%) = (number of colonies / number of seeded cells) × 100%. All experiments were performed at least three times independently.

### 2.6. Wound Healing Assay

Cells were cultured in 6-well plates until confluent monolayers were formed. A straight scratch was created in each well using a sterile 200 μL pipette tip. After washing with PBS to remove detached cells and debris, cells were incubated with serum-free medium containing the indicated drugs as mentioned above. Images of the wound area were captured at 0 h, 24 h and 48 h using an inverted microscope (Nikon, Tokyo, Japan). The migration distance was quantified using ImageJ 1.54s software (National Institutes of Health, Bethesda, MD, USA).

### 2.7. Transwell Migration Assay

Cells were trypsinized, washed with PBS, and resuspended in serum-free medium. A total of 3.8 × 10^4^ viable cells in 200 μL of serum-free medium containing the indicated drugs or DMSO were seeded into the upper chamber of a Transwell insert (8.0 μm aperture, Corning, Corning, NY, USA). The lower chamber was filled with 500 μL medium containing 10% FBS. After 24 h of incubation, non-migrated cells on the upper surface were removed with a cotton swab. Migrated cells on the lower surface were fixed with methanol for 30 min, stained with 0.5% crystal violet for 15 min, rinsed with distilled water, and air-dried. Stained cells were counted in five random fields under an inverted microscope (Nikon, Tokyo, Japan). Experiments were performed at least three times independently.

### 2.8. Matrigel Invasion Assay

For the invasion assay, the upper chamber was pre-coated with Matrigel (diluted 1:8 in serum-free DMEM, 50 μL per insert) and incubated at 37 °C for 1 h to allow gel polymerization. Subsequently, 6 × 10^4^ cells in 200 μL of serum-free medium containing the indicated drugs were added to the upper chamber. The remaining steps were the same as those for the transwell migration assay.

### 2.9. Apoptosis Detection by Flow Cytometry

MDA-MB-231 cells in the logarithmic growth phase were harvested and resuspended in DMEM to a density of 2.5 × 10^5^ cells/mL. The cell suspension (2 mL per well) was seeded into 6-well plates and cultured overnight at 37 °C in 5% CO_2_. Cells were treated according to the experimental groups for 24 h; after treatment, cells were collected. Cells were washed twice with ice-cold PBS and then stained using an Annexin V-APC/7-AAD apoptosis detection kit following the manufacturer’s instructions. Briefly, cells were resuspended in 50 μL of binding buffer containing 5 μL of 7-AAD staining solution and incubated for 5–15 min at room temperature in the dark. Subsequently, 450 μL of binding buffer was added, followed by 5 μL of Annexin V-APC, and the mixture was incubated for an additional 5–15 min at room temperature in the dark. The apoptosis rate was analyzed using a flow cytometer (Beckman Coulter, Indianapolis, IN, USA). Experiments were repeated at least three times independently.

### 2.10. Western Blot Analysis

After drug treatment for 24 h, total protein was extracted using RIPA lysis buffer supplemented with protease and phosphatase inhibitors, and protein concentration was determined using the BCA assay kit. Equal amounts of protein were separated by SDS-PAGE and transferred to PVDF membranes. Membranes were blocked with 5% BSA in TBST for 2 h at room temperature and incubated with primary antibodies against p-AKT (1:2000), p-GSK3β (1:1000), β-catenin (1:1000) and GAPDH (1:1000) overnight at 4 °C. After washing, membranes were incubated with HRP-conjugated secondary antibodies (goat anti-rabbit IgG-HRP, 1:10,000, cat. no. BA1054; goat anti-mouse IgG-HRP, 1:10,000, cat. no. BA1051, Boster, Wuhan, China) for 1 h at room temperature. Protein bands were visualized using an enhanced ECL chemiluminescence system and imaged with a ChemiDoc imaging system (Bio-Rad Laboratories, Hercules, CA, USA). Densitometric analysis was performed using ImageJ 1.54s software, and relative protein expression was normalized to GAPDH.

### 2.11. Quantitative Real-Time PCR (qPCR)

Total RNA was extracted from treated cells using TRIzol reagent according to the manufacturer’s protocol. RNA concentration and purity were assessed using a spectrophotometer, cDNA was synthesized from 1 μg of total RNA using a reverse transcription kit (HiScript II Q RT SuperMix, cat. no. R223-01, Vazyme, Nanjing, China), and qPCR was performed using SYBR master mix on an ABI Real-Time System (Applied Biosystems, Waltham, MA, USA). The mRNA expression levels of HSP90AA1 were normalized to GAPDH. The following primers were used in this study: HSP90AA1 (Forward: 5′-AGGAGGTTGAGACGTTCGC-3′; Reverse:5′-AGAGTTCGATCTTGTTTGTTCGG-3′); GAPDH (Forward:5′-ATGGGGAAGGTGAAGGTCGGAGT-3′; Reverse:5′-TAGTTGAGGTCAATGAAGGGGTC-3′). Primers were synthesized by Tsingke Biotechnology (Beijing, China).

### 2.12. Molecular Docking

Molecular docking was performed to evaluate the binding mode and affinity between baicalein and HSP90AA1. The 3D structure of baicalein was obtained from the PubChem database (PubChem CID: 5281605). The crystal structure of the human Hsp90-alpha N-domain was downloaded from the PDB database (PDB ID: 5XRB, resolution 1.65 Å). Blind molecular docking was carried out using the CB-Dock2 server (https://cadd.labshare.cn/cb-dock/ (accessed on 27 June 2026)), which automatically identifies potential binding cavities and performs docking with AutoDock Vina (version 1.2.0). A binding energy of less than −5 kcal/mol is generally considered to indicate good binding. The best-ranked docking pose was selected for further interaction analysis. Discovery Studio Visualizer v25.1.0.24284 was used to visualize the docking results and generate the 2D and 3D interaction diagrams. Only intermolecular interactions were retained for clarity.

### 2.13. Survival Analysis

The prognostic value of HSP90AA1 in TNBC was analyzed using the Kaplan–Meier Plotter online tool (https://kmplot.com/ (accessed on 8 June 2026)). TNBC patients were selected using the restrictions of ER-negative, PR-negative, and HER2-negative status. The JetSet best probe set (214328_s_at) corresponding to HSP90AA1 was selected. Patients were divided into high- and low-expression groups based on the optimal cutoff value determined by the “Auto select best cutoff” algorithm. The cutoff values were 19,479 for overall survival (OS) and 24,151 for distant metastasis-free survival (DMFS). For OS analysis, 201 patients were included (85 low expression and 116 high expression). For DMFS analysis, 424 patients were included (317 low expression and 107 high expression). OS and DMFS were assessed using the Kaplan–Meier method, and differences between the two groups were evaluated by the log-rank test, and the hazard ratio (HR) with 95% confidence interval (CI) was estimated using univariate Cox proportional hazards regression.

### 2.14. Statistical Analysis

All experiments were independently repeated at least three times. Data are presented as mean ± standard deviation (SD). Statistical analyses were performed using GraphPad Prism 11. Statistical comparisons among multiple groups were performed using one-way analysis of variance (ANOVA) followed by Tukey’s post-hoc test. The criterion for statistical significance was *p* < 0.05.

## 3. Results

### 3.1. Single Agent Cytotoxicity and Selection of Combination Concentrations

**Figure 1:**
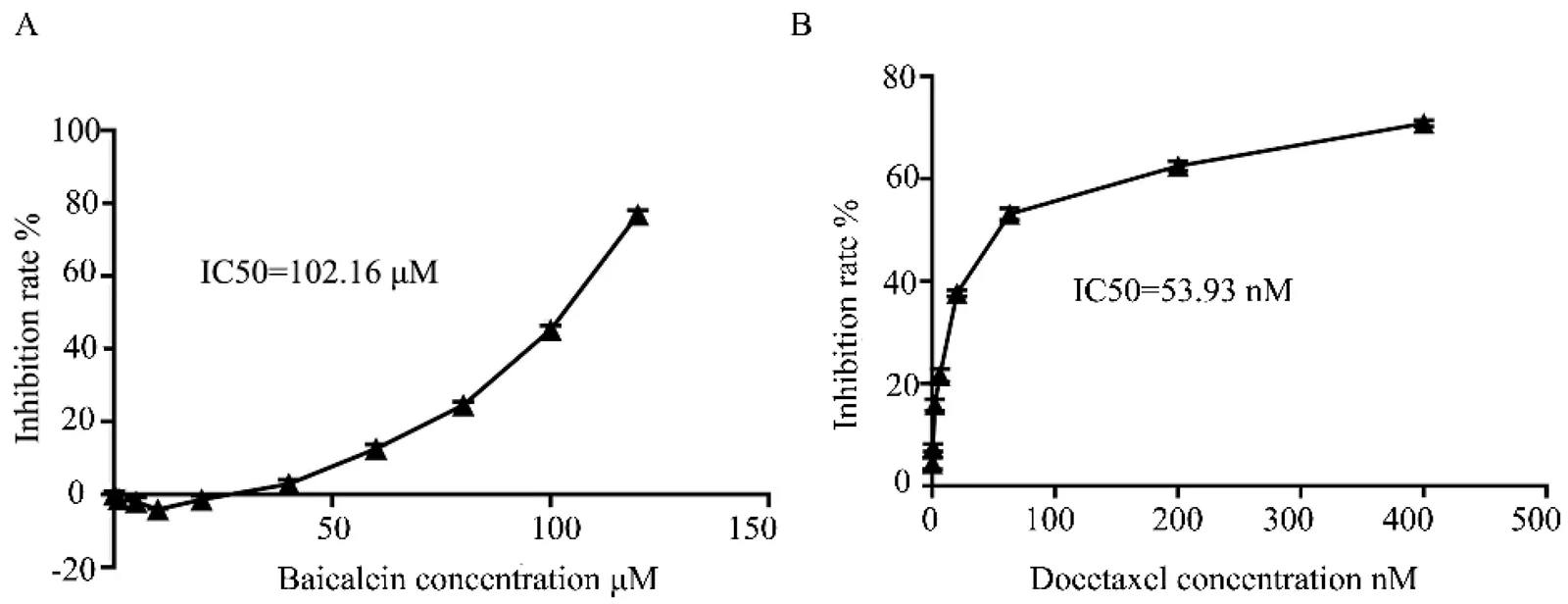
Baicalein and docetaxel inhibit MDA-MB-231 cell viability. (**A**) Concentration–response curve of baicalein. MDA-MB-231 cells were treated with the indicated concentrations of baicalein for 24 h, and cell viability was assessed. Data are presented as mean ± SD from three independent experiments. (**B**) Concentration–response curve of docetaxel. Cells were treated with docetaxel for 24 h, and viability was determined. Data are presented as mean ± SD (*n* = 3).

CCK-8 assays showed that both baicalein and docetaxel inhibited MDA-MB-231 cell viability in a concentration-dependent manner (Figure 1A,B). The IC50 values were 102.2 μM for baicalein and 53.9 nM for docetaxel. To observe potential synergy while minimizing toxicity, sub-inhibitory concentrations were selected: baicalein 80 μM and docetaxel 20 nM. The combination exhibited a potential synergistic effect with Q = 1.25 (>1.15) as determined by Jin’s Q-value method [31].

### 3.2. Pharmacological Interaction Between Baicalein and Docetaxel

**Figure 2:**
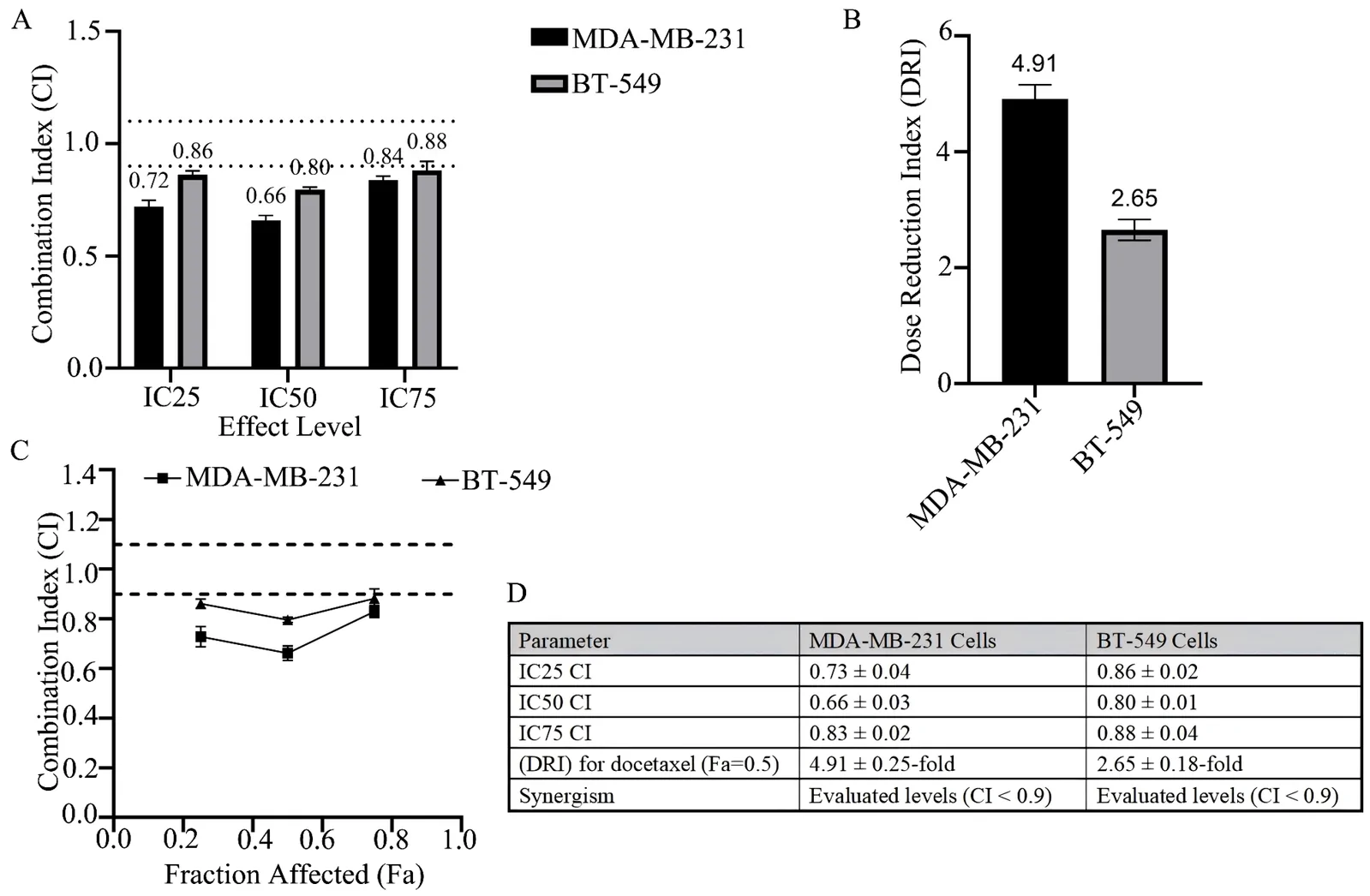
Pharmacological interaction between baicalein and docetaxel in MDA-MB-231 and BT-549 cells. Cells were treated with baicalein, docetaxel, or their combination for 24 h. The combination was prepared at a fixed numerical ratio of 4:1 (baicalein concentration in µM to docetaxel concentration in nM), corresponding to a molar ratio of 4000:1. Cell viability was assessed by the CCK-8 assay. The viability data were used to calculate the fraction affected (Fa), and CompuSyn (version 1.0) was used to determine the combination index (CI) and dose reduction index (DRI). (**A**) CI values calculated at the IC25, IC50, and IC75 effect levels for MDA-MB-231 and BT-549 cells. CI values < 0.90 indicate synergism, values from 0.90 to 1.10 indicate an additive interaction, and values > 1.10 indicate antagonism. (**B**) Model-derived DRI values for docetaxel at Fa = 0.50 in each cell line. DRI values represent descriptive comparisons of the single-agent and combination doses predicted to produce the same effect and do not indicate clinical dose reduction. (**C**) Fa–CI plot depicting the relationship between the fraction affected (Fa) and the combination index (CI). The dashed lines indicate the CI thresholds of 0.9 and 1.1. (**D**) Summary table of CI values at the specified effect levels and DRI for docetaxel. Data are presented as the mean ± SD of three independent experiments (*n* = 3). IC25 and IC50 were within the experimentally observed Fa range, whereas IC75 for docetaxel alone was extrapolated.

Given the synergistic potential suggested by Jin’s Q-value method for the selected concentrations, a Chou–Talalay median-effect analysis based on the tested fixed-ratio dilution series was performed at a fixed numerical ratio of 4:1 (baicalein in µM to docetaxel in nM; e.g., 80 µM baicalein + 20 nM docetaxel), corresponding to a molar ratio of 4000:1 (Tables S1 and S2). The median-effect parameters (Dm, m, and r) for each single drug were as follows: in MDA-MB-231 cells, baicalein exhibited a Dm of 107.69 µM, m = 1.39, and r = 0.97, whereas docetaxel exhibited a Dm of 63.04 nM, m = 0.54, and r = 0.99, the combination exhibited a Dm of 63.88 (total dose, defined as baicalein µM + docetaxel nM at a fixed 4:1 ratio), m= 0.89, and r =0.98; in BT-549 cells, the corresponding values were Dm = 87.33 µM, m = 1.20, and r = 0.99 for baicalein, and Dm = 25.52 nM, m = 0.62, and r = 0.99 for docetaxel, and Dm = 46.51 (total dose, defined as baicalein µM + docetaxel nM at a fixed 4:1 ratio), m = 0.82, and r = 0.99 for combination. All r values were above 0.95, indicating good conformity to the median-effect principle. The combination index (CI) values at IC25, IC50, and IC75 were below 0.9 in both MDA-MB-231 and BT-549 cells, indicating synergism across the evaluated effect levels (Figure 2A,C,D; Figure S1). For docetaxel alone, the IC75 level was extrapolated beyond the highest observed Fa (0.564 in MDA-MB-231 and 0.700 in BT-549), whereas IC25 and IC50 were within the experimentally observed range. The model-derived dose reduction index (DRI) for docetaxel at Fa = 0.50 was 4.91-fold in MDA-MB-231 cells and 2.65-fold in BT-549 cells (Figure 2B,D; Figure S1). Collectively, the Chou–Talalay analysis supported the synergism suggested by Jin’s Q-value method (Q = 1.25) at the tested 4:1 numerical ratio and 24-h exposure in both MDA-MB-231 and BT-549 cells.

### 3.3. Baicalein and Docetaxel Combination Potently Inhibits Long-Term Proliferation

**Figure 3:**
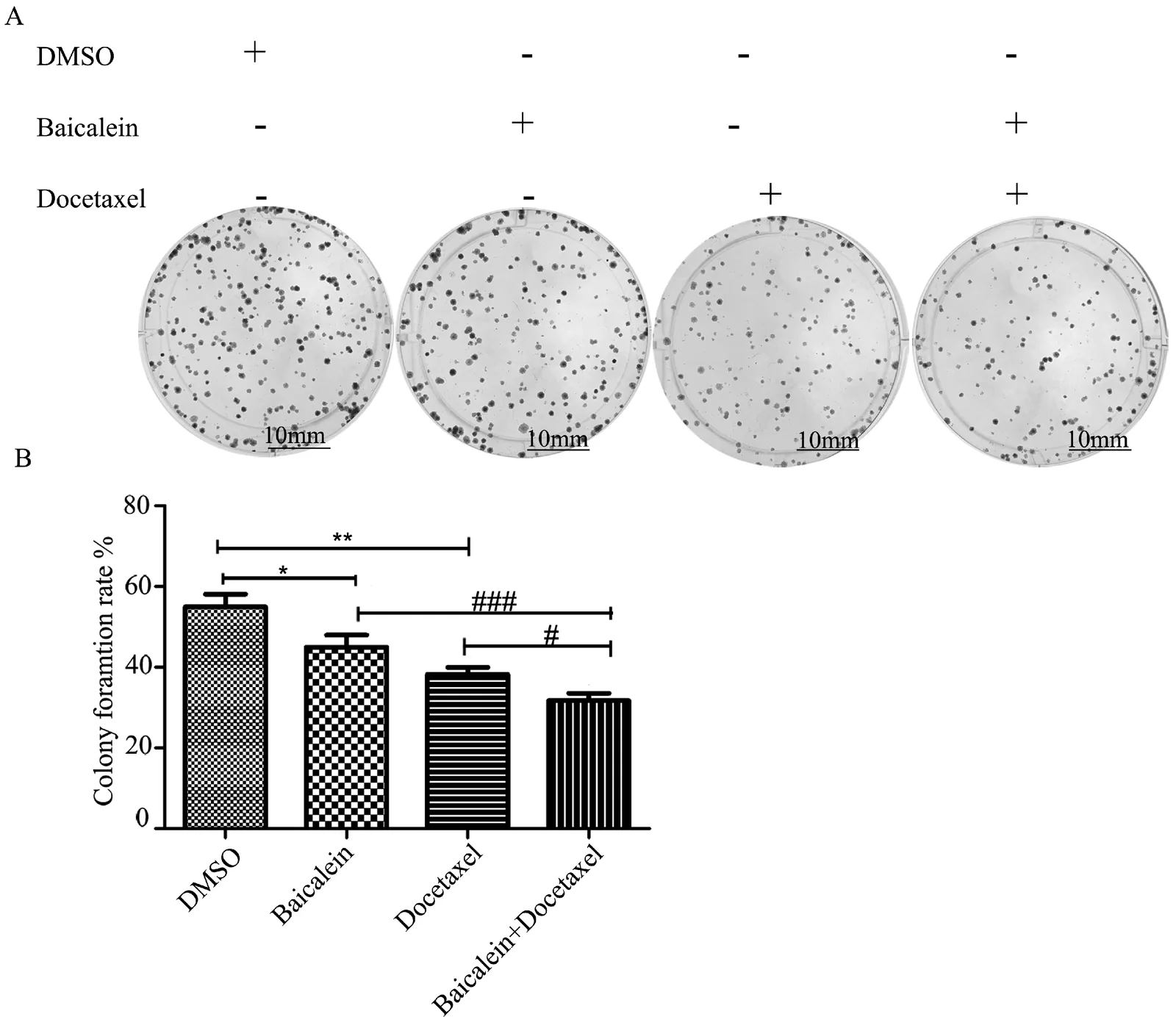
Baicalein and docetaxel combination suppresses colony formation. (**A**) Colony formation assay. Cells were treated with baicalein, docetaxel, or their combination and allowed to form colonies, then fixed and stained with crystal violet. Representative images are shown. Scale bars, 10 mm. (**B**) Quantification of colony formation. Colonies were counted and the colony formation rate was calculated. Data from three independent experiments are presented as mean ± SD. * *p* < 0.05, ** *p* < 0.01 vs. control; ### *p* < 0.001 vs. baicalein alone; # *p* < 0.05 vs. docetaxel alone.

The colony formation assay (Figure 3) revealed that baicalein or docetaxel monotherapy significantly inhibited colony formation compared with the control group (*p* < 0.05 and *p* < 0.01, respectively). Notably, the combination further reduced colony formation compared with either agent alone (*p* < 0.001 vs. baicalein; *p* < 0.05 vs. docetaxel). Consistent with the synergism confirmed by the Chou–Talalay analysis based on CCK-8 data, these results demonstrate that the combination exerts a superior inhibitory effect on long-term proliferation compared with either monotherapy.

### 3.4. Baicalein and Docetaxel Combination Potently Inhibits Cell Migration

**Figure 4:**
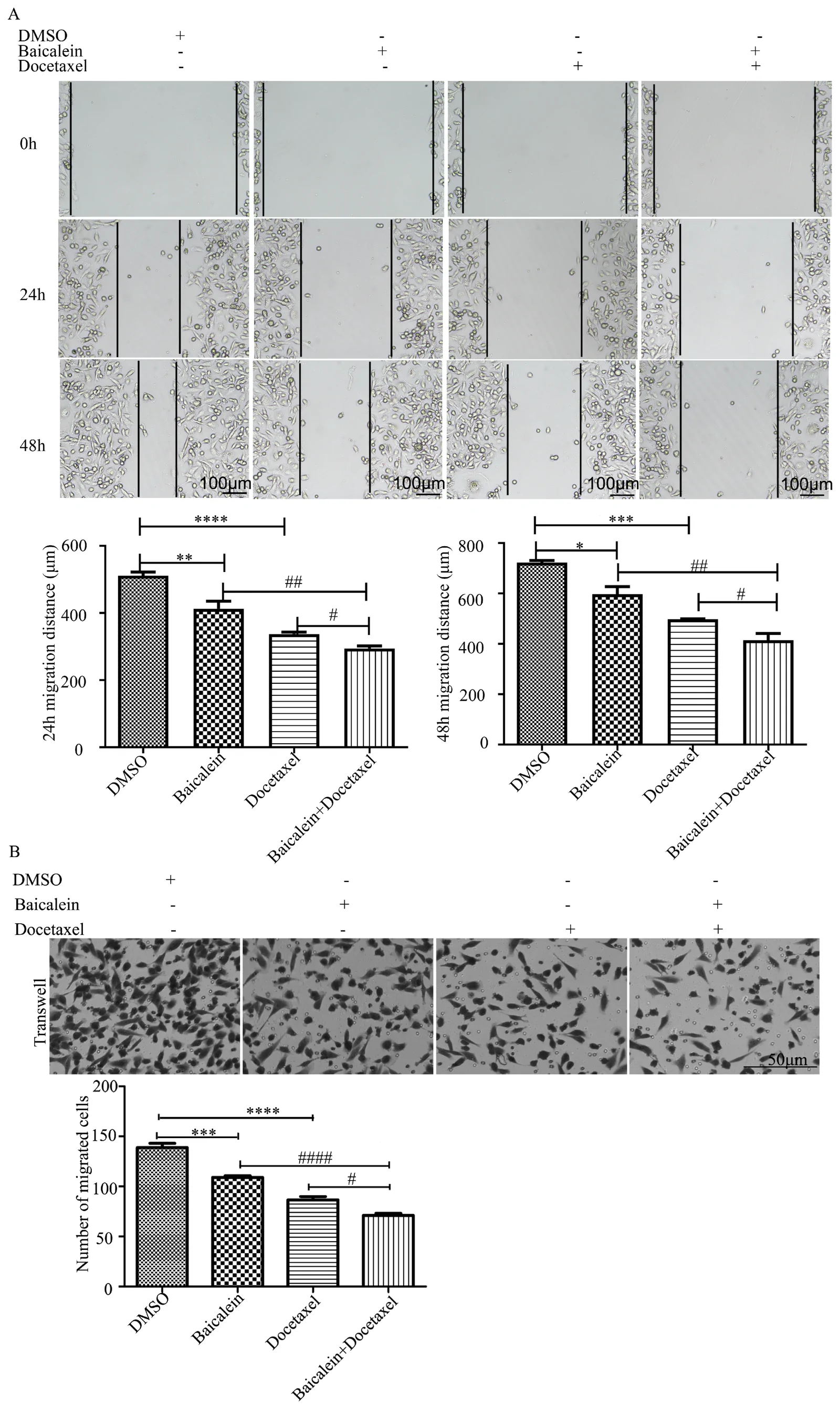
Baicalein and docetaxel combination suppresses cell migration in MDA-MB-231 cells. (**A**) Wound healing assay. Cells were treated with baicalein, docetaxel, or their combination, and wound healing was monitored at 0 h, 24 h, and 48 h. Representative images are shown. Migration distances were quantified. Scale bars = 100 µm.* *p* < 0.05, ** *p* < 0.01 *** *p* < 0.001, **** *p* < 0.0001 vs. control; # *p* < 0.05, ## *p* < 0.01 vs. corresponding monotherapy. (**B**) Transwell migration assay. Cells were treated and allowed to migrate through Transwell chambers, then fixed and stained with crystal violet. Representative images are shown. Migrated cells were quantified. Data are presented as mean ± SD from three independent experiments. *** *p* < 0.001, **** *p* < 0.0001 vs. control; # *p* < 0.05, #### *p* < 0.0001 vs. corresponding monotherapy.

The wound healing assay was performed to evaluate cell migration. As shown in Figure 4A, the migration distances were quantified at 24 h and 48 h after treatment. At both time points, baicalein alone and docetaxel alone significantly reduced migration distance compared with the control (*p* < 0.01 and *p* < 0.0001 at 24 h; *p* < 0.05 and *p* < 0.001 at 48 h). Moreover, the combination group showed a significantly shorter migration distance than baicalein alone (*p* < 0.01 at both time points) and docetaxel alone (*p* < 0.05 at both time points). These results indicate that the combination exerts a significantly greater inhibitory effect on cell migration than either monotherapy. Consistently, the Transwell migration assay (Figure 4B) showed that baicalein alone and docetaxel alone significantly reduced the number of migrated cells compared with the control group (*p* < 0.001 and *p* < 0.0001, respectively). Notably, the combination further reduced the number of migrated cells compared with baicalein alone (*p* < 0.0001) and docetaxel alone (*p* < 0.05). Collectively, these findings demonstrate that the combination of baicalein and docetaxel significantly suppresses the migratory capacity of MDA-MB-231 cells, with an efficacy superior to either agent alone.

### 3.5. Baicalein and Docetaxel Combination Potently Inhibits Cell Invasion

**Figure 5:**
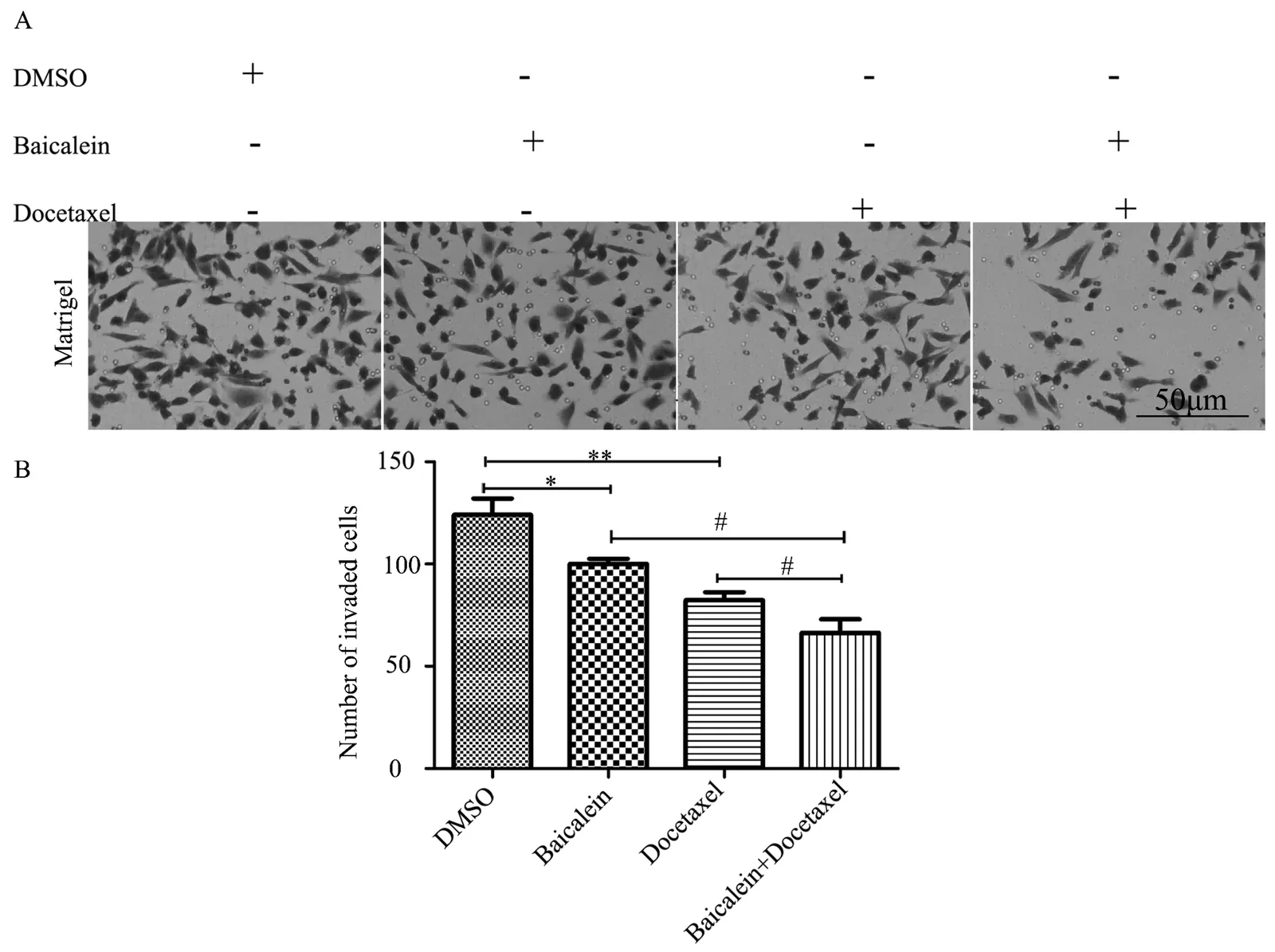
Baicalein and docetaxel combination inhibits the invasive capacity of MDA-MB-231 cells. (**A**) Matrigel invasion assay. MDA-MB-231 cells were treated with baicalein, docetaxel, or their combination, and then seeded onto Matrigel-coated transwell inserts. Invaded cells were fixed, stained, and imaged. Representative images are shown. (**B**) Quantification of invaded cells. Data from three independent experiments are presented as mean ± SD, * *p* < 0.05, ** *p* < 0.01 vs. control, # *p* < 0.05 vs. corresponding monotherapy.

The Matrigel invasion assay was performed to evaluate cell invasion. As shown in Figure 5, both baicalein alone and docetaxel alone significantly reduced the number of invasive cells compared with the control group (*p* < 0.05 and *p* < 0.01, respectively). Notably, the combination of baicalein and docetaxel further decreased the number of invasive cells, which was significantly lower than that of baicalein alone (*p* < 0.05) and docetaxel alone (*p* < 0.05). These results indicate that the combination of baicalein and docetaxel exerts a superior inhibitory effect on the invasive capacity of MDA-MB-231 cells compared with either monotherapy.

### 3.6. Baicalein and Docetaxel Combination Potently Induces Apoptosis

**Figure 6:**
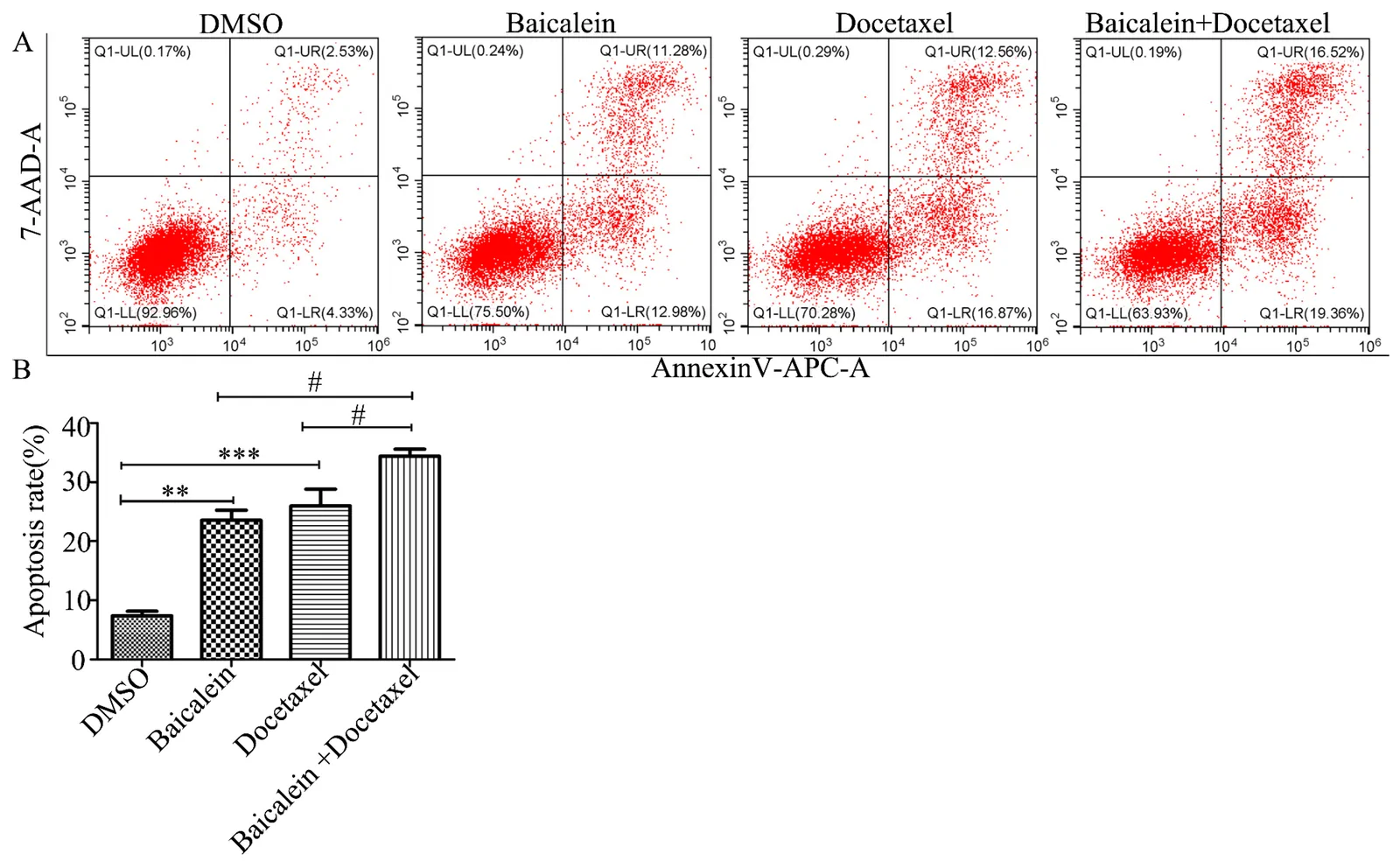
Baicalein and docetaxel combination potently induces apoptosis in MDA-MB-231 cells. (**A**) Apoptosis analysis by flow cytometry. MDA-MB-231 cells were treated with DMSO, baicalein alone, docetaxel alone, or their combination for 24 h, stained with Annexin V-APC and 7-AAD, and analyzed by flow cytometry. Representative scatter plots are shown. (**B**) Quantification of total apoptosis rate (Annexin V-positive cells). Data from three independent experiments are presented as mean ± SD in the bar graph. ** *p* < 0.01, *** *p* < 0.001 vs. control; # *p* < 0.05 vs. corresponding monotherapy.

Flow cytometry was performed to detect cell apoptosis. As shown in Figure 6, baicalein and docetaxel alone significantly increased the apoptosis rate compared with the control group (*p* < 0.01 and *p* < 0.001, respectively). Notably, the combination further elevated the apoptosis rate compared with either agent alone (both *p* < 0.05). These results indicate that the combination of baicalein and docetaxel significantly induces apoptosis in MDA-MB-231 cells, with an efficacy superior to either agent alone.

### 3.7. Baicalein and Docetaxel Combination More Effectively Suppresses the AKT/GSK3β/β-Catenin Axis Without Affecting HSP90AA1 Expression

**Figure 7:**
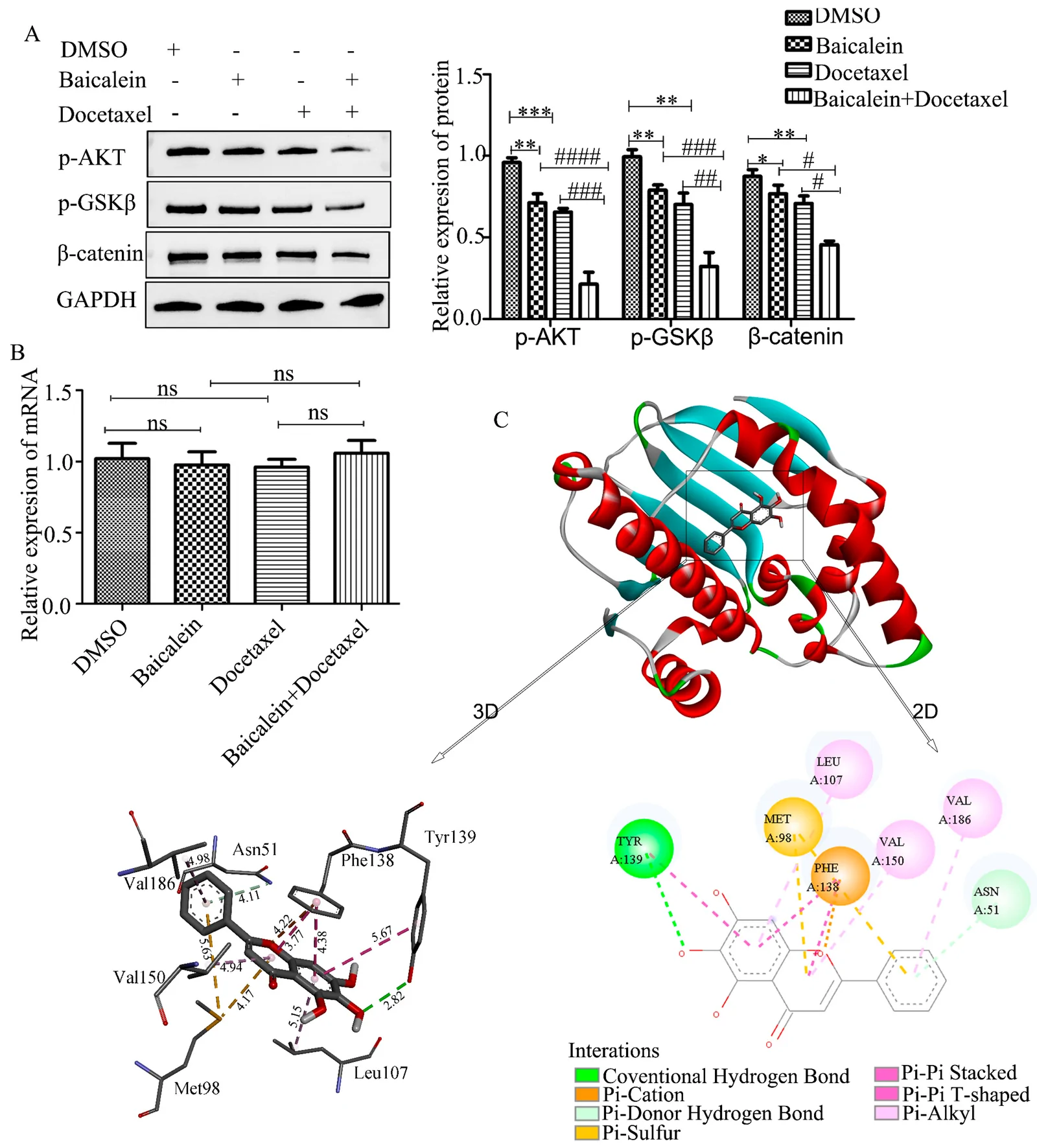
Baicalein and docetaxel combination suppresses the AKT/GSK3β/β-catenin pathway more effectively than monotherapy without affecting HSP90AA1 expression. (**A**) Western blot analysis of p-AKT, p-GSK3β, β-catenin and GAPDH. MDA-MB-231 cells were treated with DMSO, baicalein alone, docetaxel alone, or their combination. Total cellular proteins were subjected to immunoblotting using antibodies against p-AKT, p-GSK3β, β-catenin, and GAPDH. Band intensities were quantified using ImageJ 1.54s software, and signals were normalized to GAPDH. Data are presented as mean ± SD (*n* = 3). * *p* < 0.05, ** *p* < 0.01, *** *p* < 0.001 vs. control; # *p* < 0.05, ## *p* < 0.01, ### *p* < 0.001, #### *p* < 0.0001 vs. corresponding monotherapy. (**B**) qPCR analysis of HSP90AA1 mRNA expression. Total RNA was extracted from cells treated as in (**A**), and HSP90AA1 mRNA levels were quantified by qPCR and normalized to GAPDH. Data are presented as mean ± SD (*n* = 3), ns, not significant (*p* > 0.05), compared with control or compared with corresponding monotherapy. (**C**) Molecular docking of baicalein to HSP90AA1. Baicalein is shown in the binding pocket of HSP90AA1.

The AKT/GSK3β/β-catenin signaling axis is a well-established oncogenic pathway in breast cancer [14,16]. Baicalein has been reported to modulate components of this pathway, including AKT and β-catenin [23,24]. Given that our preceding results demonstrated that the baicalein–docetaxel combination potently inhibited proliferation, migration, and invasion while inducing apoptosis, we sought to determine whether these phenotypic effects were associated with modulation of the AKT/GSK3β/β-catenin axis. Furthermore, AKT is a client protein of HSP90AA1 and HSP90AA1 is known to regulate client stability and activity primarily through its chaperone cycle rather than through changes in its own expression [25,26]. Additionally, baicalein has been reported to directly bind to HSP90AA1, leading to disrupted HSP90AA1–AKT binding and reduced p-AKT [30]. Given this evidence, the baicalein–docetaxel combination may involve HSP90AA1 and its downstream AKT pathway. We therefore examined HSP90AA1 mRNA expression, assessed the AKT/GSK3β/β-catenin pathway by Western blot, and explored the binding mode of baicalein to HSP90AA1 by molecular docking. As shown in Figure 7A, compared with the control group, baicalein or docetaxel alone significantly decreased the protein levels of p-AKT (Ser473) (*p* < 0.01 and *p* < 0.001, respectively) and p-GSK3β (Ser9) (*p* < 0.05 and *p* < 0.01, respectively). Notably, the combination further reduced the levels of both phosphorylated proteins compared with either monotherapy (for p-AKT, *p* < 0.0001 vs. either agent; for p-GSK3β, *p* < 0.0001 vs. baicalein and *p* < 0.001 vs. docetaxel). Consistently, β-catenin levels were also decreased by every single agent (*p* < 0.05 and *p* < 0.01, respectively), and the combination further decreased β-catenin protein levels compared with either agent alone (both *p* < 0.05). These results indicate that the combination more effectively suppresses the AKT/GSK3β/β-catenin pathway than either agent alone. We next assessed HSP90AA1 mRNA expression by qPCR. HSP90AA1 mRNA expression did not differ significantly among groups (Figure 7B), indicating that the reduction in p-AKT was not mediated by transcriptional downregulation of HSP90AA1. To gain structural insight into the known interaction between baicalein and HSP90AA1, molecular docking was performed. Blind docking predicted five potential binding sites with Vina scores ranging from −8.9 to −5.2 kcal/mol. The top-ranked site (Cavity 1) exhibited the most favorable binding affinity and was selected for further analysis. In this site, baicalein docked into HSP90AA1, forming potential interactions with seven residues: ASN51, MET98, LEU107, PHE138, TYR139, VAL150, and VAL186. Interaction analysis showed that baicalein formed a non-covalent network with these residues, including a conventional hydrogen bond with TYR139 (2.82 Å), a Pi-donor hydrogen bond with ASN51 (4.10 Å), dual Pi-sulfur interactions with MET98 (4.17 Å and 5.63 Å), dual Pi-Pi stacked interactions with PHE138 (3.77 Å and 4.38 Å), a Pi-Pi T-shaped interaction with TYR139 (5.66 Å), and Pi-alkyl interactions with VAL150 (4.94 Å), LEU107 (5.15 Å), and VAL186 (4.98 Å) (Figure 7C). Collectively, these findings demonstrate that the combination further inhibits AKT/GSK3β/β-catenin signaling compared with monotherapy without altering HSP90AA1 mRNA. Furthermore, molecular docking predicts that baicalein binds to HSP90AA1 with a binding energy of −8.9 kcal/mol, engaging seven key residues.

### 3.8. High HSP90AA1 Expression Correlates with Poor Prognosis in TNBC

**Figure 8:**
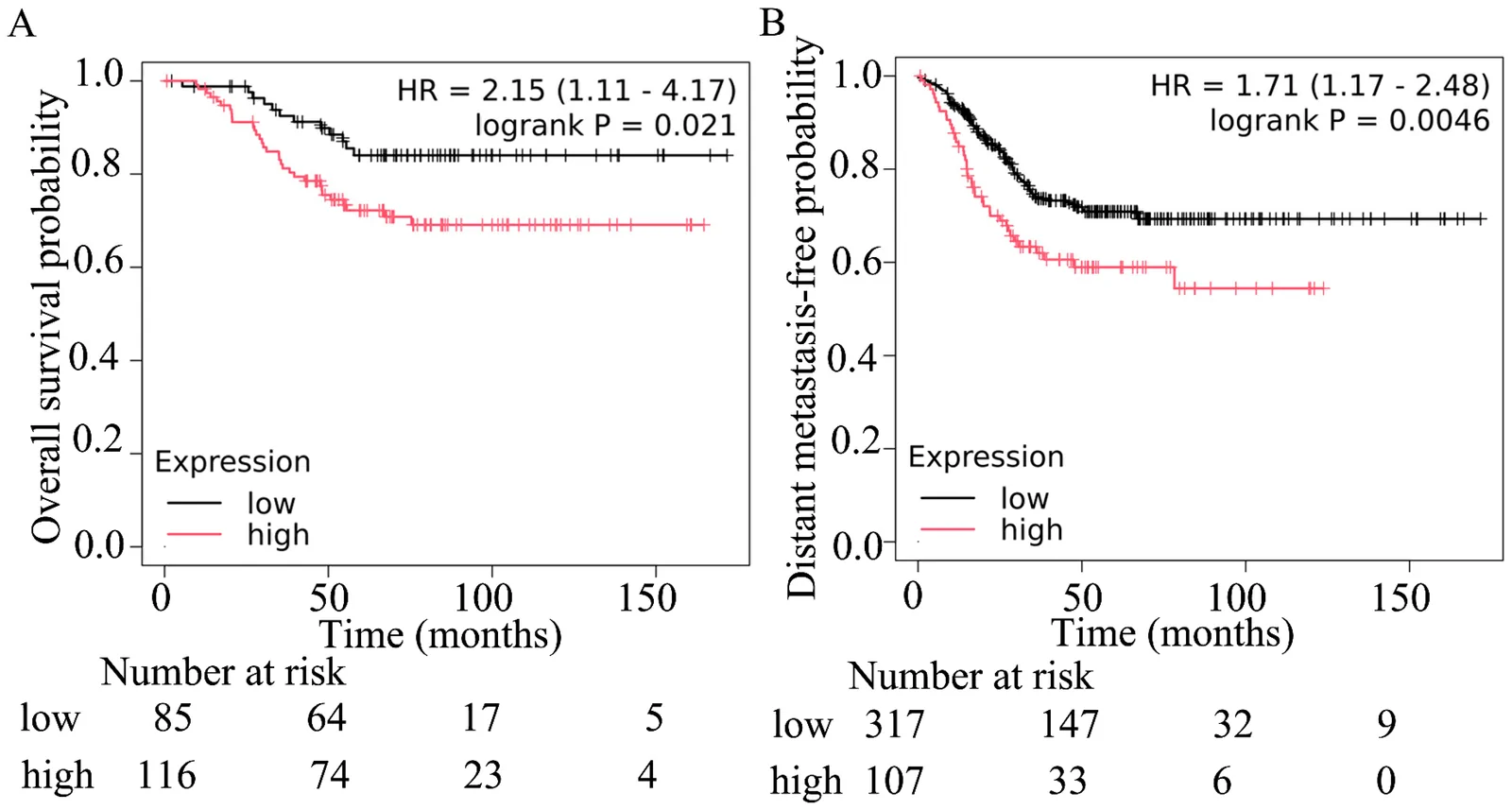
Prognostic value of HSP90AA1 expression in triple-negative breast cancer (TNBC) patients. (**A**) Overall survival (OS). Patients were stratified into low and high HSP90AA1 expression groups based on the optimal cutoff value. High HSP90AA1 expression was associated with significantly shorter OS (HR = 2.15, 95% CI: 1.11–4.17, logrank *p* = 0.021). (**B**) Distant metastasis-free survival (DMFS). Patients were stratified into low and high HSP90AA1 expression groups based on the optimal cutoff value. High HSP90AA1 expression was associated with significantly worse DMFS (HR = 1.71, 95% CI: 1.17–2.48, logrank *p* = 0.0046). The upper quartile survival for DMFS was 34.346 months in the low expression group and 17.31 months in the high expression group. Data were analyzed using the Kaplan–Meier Plotter online tool.

The preceding results demonstrated that the baicalein–docetaxel combination more effectively suppressed the AKT/GSK3β/β-catenin signaling axis than either agent alone without affecting HSP90AA1 mRNA expression in TNBC cells, and molecular docking revealed a potential direct interaction between baicalein and HSP90AA1. To bridge these findings with patient outcomes, we evaluated the prognostic significance of HSP90AA1 in TNBC using the Kaplan–Meier Plotter database. We focused on two clinical endpoints that directly reflect the observed phenotypes: overall survival (OS) and distant metastasis-free survival (DMFS). As shown, TNBC patients with high HSP90AA1 expression had significantly shorter overall survival compared with those with low expression (logrank *p* = 0.021, Figure 8A). Moreover, high HSP90AA1 expression was also significantly associated with worse distant metastasis-free survival (DMFS) (logrank *p* = 0.0046, Figure 8B). These results demonstrate that high HSP90AA1 expression is a risk factor for poor prognosis in TNBC, highlighting its prognostic significance in this aggressive subtype.

## 4. Discussion

In this study, the synergistic effect of baicalein and docetaxel was evaluated in MDA-MB-231 and BT-549 cells, and subsequent functional and mechanistic investigations were performed in MDA-MB-231 cells. Our results demonstrated that the combination more effectively inhibited proliferation, migration, and invasion than either agent alone while promoting apoptosis, accompanied by suppression of the AKT/GSK3β/β-catenin pathway without altering HSP90AA1 mRNA expression. Molecular docking further suggested that baicalein binds to HSP90AA1, and survival analysis indicated that high HSP90AA1 expression is associated with poor prognosis in TNBC.

The limited efficacy of docetaxel is multifactorial, with aberrant activation of the AKT signaling pathway being a key contributor [8,32]. In our study, the potent inhibition of p-AKT (Ser473) by the combination in MDA-MB-231 cells was associated with the observed enhancement of anti-malignant phenotypes. Notably, previous research in RAW264.7 cells has not only confirmed the baicalein–HSP90 interaction using multiple biological assays, including Surface Plasmon Resonance (SPR), Microscale Thermophoresis (MST), and Fluorescence Quenching Assay (FQA), but also demonstrated via co-localization and Co-immunoprecipitation (Co-IP) that baicalein disrupts the binding between HSP90 and its client protein AKT, leading to AKT destabilization and inactivation [30,33]. Consistently, our molecular docking analysis revealed that baicalein could bind within the active pocket of HSP90AA1 (ASN51, MET98, LEU107, PHE138, TYR139, VAL150, VAL186). Importantly, sequence alignment and existing literature confirm that these key residues constitute the highly conserved ATP-binding pocket of HSP90AA1 [33], which is in agreement with independent docking studies using different HSP90AA1 crystal structures [34,35]. Furthermore, our qPCR results showed that HSP90AA1 mRNA expression did not differ significantly among the treatment groups, suggesting that baicalein may not downregulate HSP90AA1 at the transcriptional level. This observation is consistent with independent findings. Indeed, studies have demonstrated that baicalein directly targets the ATPase domain of HSP90 to inhibit its chaperone function without altering the HSP90 protein levels [33], and that compounds disrupting HSP90–AKT binding similarly reduce p-AKT while leaving HSP90 protein expression unchanged [36]. Functionally, HSP90AA1 acts as a molecular chaperone for AKT [30,37], exerting dual protection: on the one hand, it maintains the correct conformation of AKT to prevent its degradation, thereby ensuring the availability of total AKT; on the other hand, it directly interacts with AKT to protect its phosphorylated (p-AKT) state [27,37,38]. When the chaperone function of HSP90AA1 is inhibited, both protective pathways are disrupted, ultimately leading to a marked decrease in p-AKT levels.

Based on these findings, in MDA-MB-231 cells, it is reasonable to suggest that baicalein inhibits HSP90AA1 function, which could be associated with reduced p-AKT levels and consequently with reduced AKT signaling activity. The reduction in p-AKT was accompanied by decreased phosphorylation of GSK3β at Ser9 (increased GSK3β activity), which in turn phosphorylates β-catenin and promotes its degradation [17,22]. Consequently, β-catenin protein levels decline. In line with this, our results showed that the combination further reduced p-AKT, p-GSK3β, and β-catenin protein levels compared with either agent alone, consistent with more effective modulation of the AKT/GSK3β/β-catenin axis. Given that β-catenin drives the transcription of genes involved in proliferation, survival, and metastasis [39,40,41,42], its downregulation provides a plausible association between suppression of the AKT/GSK3β/β-catenin axis and the observed anti-malignant phenotypes, including reduced proliferation, migration, invasion, and enhanced apoptosis. For migration and invasion, although the serum-free upper chamber and 24-h incubation minimize the influence of proliferation, a partial contribution of reduced viability to the observed reduction in migration and invasion cannot be fully excluded.

Known HSP90AA1 inhibitors target its function, leading to decreased p-AKT levels [43,44,45]. In line with the reported disruption of HSP90AA1–AKT binding by baicalein in RAW264.7 cells, the MST assay in that study further confirmed that baicalein competitively binds to the ATP-binding pocket of HSP90 [30],and given that our docking predicts baicalein binds to the conserved ATP-binding pocket of HSP90AA1, we speculate that baicalein may act in a similar ATP-competitive manner to disrupt the HSP90AA1–AKT interaction in TNBC cells; however, whether baicalein disrupts the HSP90AA1–AKT interaction in TNBC cells remains to be determined by Co-IP and co-localization. Moreover, direct binding between baicalein and HSP90AA1 requires further confirmation by biophysical assays such as CETSAs, SPR, or MST in TNBC cells.

Consistent with this notion, the survival analysis provides prognostic context for the above mechanism. High HSP90AA1 expression predicts poor prognosis in TNBC, which is consistent with previous reports [46,47]. However, this prognostic association does not independently validate the proposed drug mechanism. It remains a hypothesis that tumors with high HSP90AA1 expression may be more dependent on its function to maintain the activity of pro-survival kinases such as AKT, potentially leading to higher p-AKT levels. Regarding the Chou–Talalay analysis, the DRI values for docetaxel in tested cells are model-derived estimates based on the tested fixed-ratio dilution series. These values suggest a potential dose-reduction effect in vitro but do not represent demonstrated clinical dose reduction or therapeutic benefit. Collectively, these considerations indicate that the in vitro synergy observed in this study should not be directly extrapolated to clinical efficacy.

Several limitations should be acknowledged. First, although the synergistic effect was validated in MDA-MB-231 and BT-549 cells, the functional and mechanistic experiments were performed in MDA-MB-231 cells and no in vivo experiments were performed. TNBC is heterogeneous, and findings from MDA-MB-231 cells may not fully represent all subtypes. Nevertheless, published evidence from other models suggests the possibility of broader relevance of our findings: consistent with the synergistic effects observed in MDA-MB-231 and BT-549 cells, synergistic effects of baicalein and docetaxel have also been reported in MCF-7, non-small cell lung cancer, and anaplastic thyroid cancer [13,24,48]. Furthermore, the anti-TNBC activity of baicalein has been validated in additional TNBC cell lines, including BT-549, Hs578T, and MDA-MB-468 [12,49], and the AKT/GSK3β/β-catenin pathway has been confirmed as a key target of baicalein [50,51]. Although the functional and mechanistic experiments were performed only in MDA-MB-231 cells, our findings are consistent with published evidence from other TNBC models and diverse cancer types. Nevertheless, the interaction should be described as observed in the two tested TNBC cell lines under the specified conditions, and key functional and signaling findings should also be validated in BT-549 cells if broader mechanistic conclusions are retained. Regarding in vivo validation, the lung cancer study showed that combination therapy reduced tumor volume and weight without additional toxicity, supporting potential translatability [24]. Nonetheless, further in vivo studies are warranted. Second, although HSP90AA1 mRNA levels were unchanged, HSP90AA1 protein levels were not directly examined. Consequently, we cannot fully exclude the possibility that the observed reduction in p-AKT and downstream signaling is caused by altered HSP90AA1 protein levels. Nevertheless, we hypothesize that baicalein may modulate HSP90AA1 function. This hypothesis is based on the following considerations. First, the reported competitive binding of baicalein to the ATP-binding pocket of HSP90 and our molecular docking results showing that the interacting residues reside within this pocket suggest possible functional regulation. Second, published evidence from other systems suggests that baicalein can affect HSP90 chaperone function without altering HSP90 protein levels [33]. In addition, our qPCR results showed unchanged HSP90AA1 mRNA levels, indicating that no significant transcriptional regulation was detected under these conditions. However, unchanged mRNA levels do not exclude changes in protein abundance, stability, localization, or activity and therefore do not by themselves distinguish between functional and expression-level regulation. Therefore, HSP90AA1 should be considered a proposed target rather than a confirmed mediator. Direct examination of HSP90AA1 protein levels and functional validation in TNBC cells is still warranted. Third, rescue experiments (e.g., overexpression of constitutively active AKT) are needed to prove that AKT is an essential node mediating the combination effect. Although a previous study demonstrated that an HSP90-targeting compound disrupted HSP90–AKT binding, reduced p-AKT levels and suppressed tumorigenesis, overexpression of constitutively active AKT restored p-AKT and abrogated the effects of the HSP90 inhibitor [36]. Together with the HSP90 chaperone function discussed above, these findings support the notion that AKT is a critical downstream mediator of HSP90 inhibition. However, given the differences in cancer type and compound, direct rescue experiments in our TNBC model are still required to confirm AKT as the essential mediator of the combination effect. Fourth, as discussed above, although our molecular docking data and the literature collectively point to the binding of baicalein to HSP90AA1, and studies further demonstrate that this binding disrupts the HSP90AA1–AKT interaction and downregulates p-AKT [30,33], these findings cannot be fully extrapolated to TNBC cells due to potential cell-type-specific differences. Therefore, further biological validation using approaches such as SPR, cellular thermal shift assays (CETSA), or (co-IP [30,33] in our TNBC model is still warranted to confirm these interactions. Fifth, pharmacokinetic data show that plasma docetaxel concentrations post-infusion range from approximately 18.2 to 464 nM [52], indicating that the 20 nM docetaxel concentration used in our in vitro study is within the range of clinically achievable plasma concentrations. For baicalein, although the concentration used here (80 μM) falls within the reported IC50 range (20–200 μM) [53], it is uncertain whether such concentrations can be achieved in plasma after oral administration because of the poor oral bioavailability of baicalein. Achieving effective intratumoral concentrations will therefore require strategies to improve bioavailability and tumor targeting. Recent advances in nanoparticle-based delivery systems may offer a promising approach to overcome this limitation [54]. However, future in vivo pharmacokinetic and efficacy studies are needed to determine whether these systems can reproduce the observed combination benefit. Despite these limitations, our study provides initial evidence of a synergistic interaction between baicalein and docetaxel in TNBC and suggests a possible chemosensitization mechanism that warrants further investigation.

In conclusion, baicalein combined with docetaxel exhibits synergistic effects in both MDA-MB-231 and BT-549 cells under the tested fixed-ratio and 24-h exposure conditions. In MDA-MB-231 cells, this combination more effectively inhibits proliferation, migration, and invasion than either agent alone, and promotes apoptosis. This enhanced activity is associated with suppression of the AKT/GSK3β/β-catenin signaling axis and occurs without downregulating HSP90AA1 mRNA expression. Molecular docking suggested an interaction between baicalein and HSP90AA1. Combining these docking-identified residues with literature evidence raises the possibility that binding occurs within the ATP-binding pocket of HSP90AA1. Together with previous evidence that baicalein targets HSP90AA1, disrupts HSP90AA1–AKT interaction, and downregulates p-AKT, these findings suggest that the chaperone function of HSP90AA1 may be affected. The prognostic significance of HSP90AA1, combined with our in vitro findings, suggests its potential as a candidate target for TNBC. However, conclusive preclinical translation requires further functional and mechanistic validation in additional TNBC cell lines and in vivo models.

## Data Availability

The original contributions presented in this study are included in the article. Further inquiries can be directed to the corresponding author.

## Supplementary Materials

The following supporting information can be downloaded at: https://www.mdpi.com/article/10.3390/cimb48101066/s1.

## Abbreviations

The following abbreviations are used in this manuscript:

TNBC	Triple-Negative Breast Cancer
HSP90AA1	Heat Shock Protein 90 Alpha family class A member 1
AKT	Protein Kinase B
GSK3β	Glycogen Synthase Kinase 3β
β-catenin	Catenin Beta-1
GAPDH	Glyceraldehyde-3-Phosphate Dehydrogenase
OS	Overall Survival
DMFS	Distant Metastasis-Free Survival
SPR	Surface Plasmon Resonance
CETSAs	Cellular Thermal Shift Assays
co-IP	Co-immunoprecipitation
ER	Estrogen Receptor
PR	Progesterone Receptor
HER2	Human Epidermal Growth Factor Receptor 2
MST	Microscale Thermophoresis
FQA	Fluorescence Quenching Assay
CI	Combination Index
DRI	Dose Reduction Index
Fa	Fraction affected
