# Supplementary material for: Baicalein Synergizes with Docetaxel in TNBC and the Combination Suppresses AKT/GSK3β/β-Catenin Signaling
Source: Curr Issues Mol Biol. 2026 Oct 15;48(10):1066. doi: 10.3390/cimb48101066 (PMC13703623; doi:10.3390/cimb48101066)
Supplement: Supplementary file 1 [file cimb-48-01066-s001.zip › Supplementary Figure.pdf]

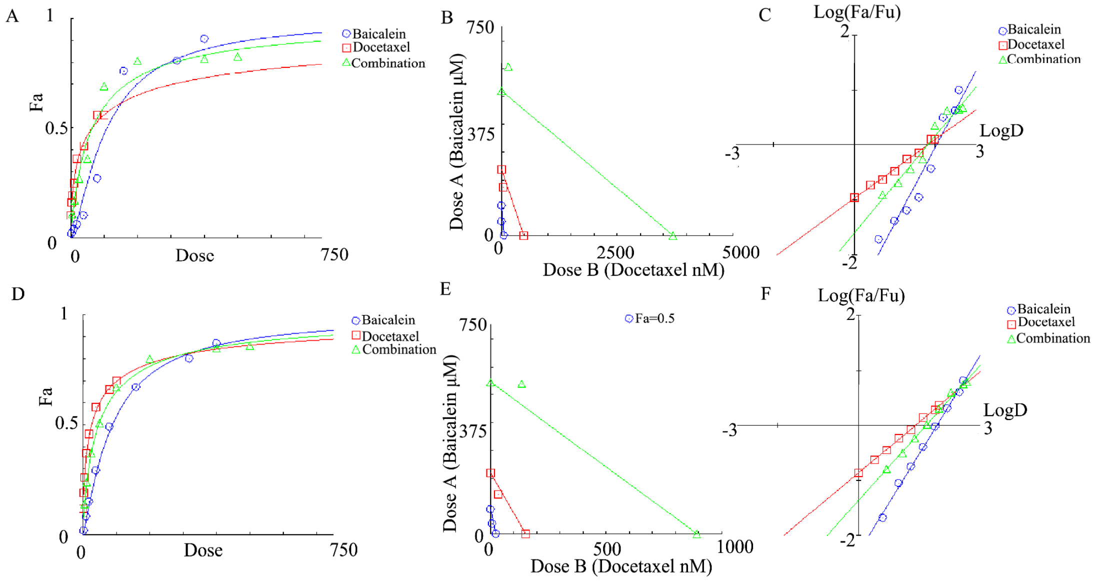

Figure S1. Chou–Talalay analysis of baicalein and docetaxel combination in MDA-MB-231 and BT-549 cells. **(A–C)** MDA-MB-231 cells; **(D–F)** BT-549 cells. **(A, D)** Dose–effect curves for baicalein (Baicalein, blue circles), docetaxel (Docetaxel, red squares), and their combination (Combination, green triangles). The combination was tested at a fixed 4:1 ratio (baicalein in μM to docetaxel in nM; e.g., 80 μM baicalein + 20 nM docetaxel). This numerical ratio corresponds to a molar ratio of 4000:1 (e.g., 80 μM baicalein = 80,000 nM; 80,000 nM : 20 nM = 4000:1). For the combination, the dose axis represents total dose, defined as baicalein [μM] + docetaxel [nM]. Data points are mean fraction affected (Fa) from three independent experiments; curves were fitted using the median-effect equation in CompuSyn (version 1.0). **(B, E)** Isobolograms at Fa = 0.5 (blue circles), 0.75 (red squares), and 0.9 (green triangles). The X-axis represents docetaxel concentration (nM) and the Y-axis represents baicalein concentration (μM). The straight lines connecting the single-agent doses indicate additivity; points below these lines indicate synergism. **(C, F)** Median-effect plots (log(Fa/Fu) vs. log(Dose)) for baicalein, docetaxel, and their combination. Dm for the combination is expressed as total dose (baicalein [μM] + docetaxel [nM]) at the fixed 4:1 ratio. CI values were calculated using CompuSyn; CI < 0.9 indicates synergism, 0.9–1.1 additivity, and > 1.1 antagonism.
