# Supplementary material for: Baicalein Synergizes with Docetaxel in TNBC and the Combination Suppresses AKT/GSK3β/β-Catenin Signaling
Source: Curr Issues Mol Biol. 2026 Oct 15;48(10):1066. doi: 10.3390/cimb48101066 (PMC13703623; doi:10.3390/cimb48101066)
Supplement: Supplementary file 1 [file cimb-48-01066-s001.zip › Supplementary Tables.pdf]

Table S1. Dose–response data for baicalein, docetaxel, and their combination in MDA-MB-231 cells

Treatment	Concentration	Fa1	Fa2	Fa3	Mean
Baicalein	4 µM	0.010	0.012	0.034	0.02
	10 µM	0.030	0.028	0.060	0.04
	20 µM	0.040	0.045	0.080	0.06
	40 µM	0.080	0.075	0.150	0.10
	80 µM	0.268	0.275	0.260	0.27
	160 µM	0.800	0.785	0.710	0.77
	320 µM	0.814	0.825	0.805	0.82
	400 µM	0.910	0.905	0.910	0.91
Docetaxel	1 nM	0.098	0.095	0.100	0.10
	2.5 nM	0.161	0.155	0.167	0.16
	5 nM	0.193	0.200	0.185	0.19
	10 nM	0.246	0.240	0.250	0.25
	20 nM	0.359	0.365	0.354	0.36
	40 nM	0.418	0.430	0.420	0.42
	80 nM	0.555	0.560	0.575	0.56
	100 nM	0.563	0.570	0.558	0.56
Combination (4:1, BAI µM : DTX nM)	4 µM BAI + 1 nM DTX	0.107	0.105	0.121	0.11
	10 µM BAI + 2.5 nM DTX	0.175	0.170	0.157	0.17
	20 µM BAI + 5 nM DTX	0.258	0.265	0.282	0.27

Treatment	Concentration	Fa1	Fa2	Fa3	Mean
	40 µM BAI + 10 nM DTX	0.290	0.395	0.381	0.36
	80 µM BAI + 20 nM DTX	0.743	0.675	0.660	0.69
	160 µM BAI + 40 nM DTX	0.810	0.815	0.803	0.81
	320 µM BAI + 80 nM DTX	0.819	0.816	0.827	0.82
	400 µM BAI + 100 nM DTX	0.825	0.830	0.821	0.83

Fa, fraction affected (Fa = 1 − relative cell viability). Data are from three independent experiments. For the combination, the fixed numerical ratio 4:1 refers to baicalein concentration in µM to docetaxel concentration in nM (e.g., 80 µM baicalein + 20 nM docetaxel) This numerical ratio corresponds to a molar ratio of 4000:1 (e.g., 80 µM baicalein = 80,000 nM; 80,000 nM : 20 nM = 4000:1). Mean was calculated from three independent experiments. These data were used for Chou–Talalay analysis; median-effect parameters (Dm, m, r) were derived from the averaged Fa values, while CI and DRI values were calculated separately for each independent experiment and are reported as mean ± SD in the main text.

Table S2. Dose–response data for baicalein, docetaxel, and their combination in BT-549 cells

Treatment	Concentration	Fa1	Fa2	Fa3	Mean
Baicalein	4 µM	0.030	0.025	0.018	0.02
	10 µM	0.080	0.075	0.071	0.08
	20 µM	0.160	0.154	0.150	0.16
	40 µM	0.300	0.295	0.289	0.30
	80 µM	0.500	0.492	0.487	0.49
	160 µM	0.700	0.629	0.692	0.67
	320 µM	0.840	0.736	0.833	0.80
	400 µM	0.870	0.862	0.864	0.87

Treatment	Concentration	Fa1	Fa2	Fa3	Mean
Docetaxel	1 nM	0.130	0.125	0.117	0.12
	2.5 nM	0.200	0.191	0.187	0.19
	5 nM	0.270	0.261	0.257	0.26
	10 nM	0.383	0.376	0.370	0.38
	20 nM	0.470	0.462	0.459	0.46
	40 nM	0.588	0.580	0.578	0.58
	80 nM	0.670	0.663	0.661	0.67
	100 nM	0.700	0.709	0.692	0.70
Combination (4:1, BAI µM : DTX nM)	4 µM BAI + 1 nM DTX	0.150	0.140	0.137	0.14
	10 µM BAI + 2.5 nM DTX	0.250	0.241	0.236	0.24
	20 µM BAI + 5 nM DTX	0.380	0.373	0.368	0.37
	40 µM BAI + 10 nM DTX	0.520	0.513	0.509	0.51
	80 µM BAI + 20 nM DTX	0.680	0.673	0.670	0.67
	160 µM BAI + 40 nM DTX	0.800	0.794	0.793	0.80
	320 µM BAI + 80 nM DTX	0.860	0.845	0.855	0.85
	400 µM BAI + 100 nM DTX	0.870	0.847	0.864	0.86

Table note: same as Table S1.
